# A novel temporary hybrid mental health service in Brazil during the COVID-19 pandemic

**DOI:** 10.47626/2237-6089-2022-0472

**Published:** 2023-10-31

**Authors:** Marcelo Bruno Generoso, Amanda Menon Pelissoni, Ana Alice Freire de Sousa, Raquel do Lago Favaro, Paulo Mazaferro, Mariana Bendlin Calzavara, Andrea de Abreu Feijó de Mello

**Affiliations:** 1 Secretaria Municipal da Saúde Prefeitura da Cidade de São Paulo São Paulo SP Brazil Secretaria Municipal da Saúde, Prefeitura da Cidade de São Paulo, São Paulo, SP, Brazil.; 2 Faculdade de Ciências Médicas Santa Casa de São Paulo São Paulo SP Brazil Faculdade de Ciências Médicas, Santa Casa de São Paulo, São Paulo, SP, Brazil.; 3 Faculdade Israelita de Ciências da Saúde Albert Einstein São Paulo SP Brazil Faculdade Israelita de Ciências da Saúde Albert Einstein, São Paulo, SP, Brazil.; 4 Hospital Israelita Albert Einstein São Paulo SP Brazil Hospital Israelita Albert Einstein, São Paulo, SP, Brazil.; 5 Universidade Federal de São Paulo São Paulo SP Brazil Universidade Federal de São Paulo, São Paulo, SP, Brazil.

As elegantly published by Tergolina et al.,^1^ in Brazil, public mental health care is organized on a network basis, focused on outpatient facilities with special units depending on level of complexity.^2^ These include the Centers for Psychosocial Attention (CPSA), each of which has an interdisciplinary team of social workers, psychologists, nurses, occupational therapists and psychiatrists to provide intensive treatment for patients with functional impairment due to severe and chronic mental disorders. The results published by Tergolina et al. showed that treatment adherence may be a challenge at these facilities.^1^

The COVID-19 pandemic added an additional burden to this challenge, overloading healthcare systems and compelling public administrators to redirect resources and staff to the pandemic frontline and convert inpatient psychiatry units located in general hospitals into COVID-19 units.^3,4^ Although understandable, this strategy reduced available beds for patients in need of hospitalization due to mental illness.

Faced with an overwhelming number of patients with respiratory symptoms, two hospital inpatient psychiatry units in São Paulo were converted into COVID-19 units. To tackle the issue of reduction of psychiatric beds, the Sao Paulo Municipal Health Department took a bold approach and set up a novel temporary hybrid mental health service for these extreme times.

This novel hybrid facility was implemented within the existing structure of the Paraisopolis CPSA after a refit had been conducted to enable adequate admission of patients in need of hospitalization, including construction of two separate inpatient units, for patients with and without respiratory symptoms, and sanitary installations, and workforce expansion and implementation of night shifts and a proper legal process for involuntary admission for treatment.

Some interesting effects were perceived, such as highly humanized care provided by staff specialized in managing severely ill patients in an outpatient setting and only resorting to hospitalization as a last option, a high sense of importance accorded to patients’ needs and individuality, strong patient-staff bonds that facilitated follow-up treatment in the same unit and the possibility that, during their hospital stay, patients could take part in other activities beyond the inpatient unit under staff supervision, speeding up the rehabilitation process. Additionally, it was also notable that staff were highly committed to the essence of the CPSA, never forgetting that the continuous improvement of mental healthcare in Brazil is a one-way process.

Additionally, this temporary unit also provided a great learning opportunity for medical interns and psychiatry residents who were included in its daily routine and reinforced its medical workforce. This novel facility offered a unique learning experience in a multidisciplinary and humanized environment that, it is believed, reduced stigma and benefitted medical training during the pandemic.

At first glance, setting up an inpatient unit inside a CPSA established during the Brazilian Psychiatry Reform process may seem strange and could have sparked off a heated debate, but in times when adaptations were needed, new ways of doing things became the standard in everyone’s lives, and it was no different at this facility, where healthcare workers came together to aid patients in need.
